# Hemiopia

**Published:** 1877-07

**Authors:** 


### HEMIOPIA.

This is a condition of the eyes in which a person is able to see but the half of any object. The obliterated portion being to the right or left, rarely at the top or bottom. In looking into a person's face, the subject of this malady sees but one eye, or but one half of the face. In attempting to read a sign over a door, for instance, as JOHNSON, it would appear as SON—the first half of the name being invisible. In this example, the loss of sight would be to the left, and the left half of every object would be entirely missing. Sometimes, the defect exists only in the one eye, but more frequently, it matters not which eye is tested, the omission remains the same in the object contemplated. Sometimes the absent portion of the object is not veiled in complete darkness, but may be of a shaded darkness, in which the outline is faintly traced.

The patient mentally refers the blotted-out appearance to external impressions, as if something were held before the eyes and could be wiped away. Frequently, they are found wiping at the eyes as though some deposit of mucus were interfering with the perfect function of the organ.

This defect in vision is the result of a partial paralysis of the *retina*, which may be engendered in a variety of ways. When the darkness is but momentary, passing away in a few minutes, or hours, it is likely to be occasioned by indigestion, dispepsia, or other difficulty of the stomach. We have seen a person who has had this defect in the vision of both eyes, now and then, for ten years, lasting but a couple of hours each time, but always occasioning the greatest alarm. In his case, it was always the sure forerunner of a severe fit of indigestion, the eye symptoms promptly disappearing with the relief from the stomach ailment.

Other cases have come under our observation which could be traced to some disturbing cause in the general system, as fatigue, severe mental strain, or other bodily ailment, the unpleasant eye-symptoms always disappearing when the patient had been restored to health.

But, now and then, a case occurs in which the dimness of vision remains in spite of all treatment directed toward the restoration of health. Such cases either indicate some serious lesion in the brain or optic nerve, or a dangerous paralysis of the *retina*. The symptoms are always of a sufficiently alarming character to warrant the patient in seeking early competent medical advice, ere the paralysis becomes complete and the eyes hopelessly blind.

